# ALTERNATIVE FISTULA RISK SCORE AND FIRST POSTOPERATIVE DAY DRAIN FLUID AMYLASE AS PREDICTORS OF PANCREATIC FISTULA AFTER PANCREATICODUODENECTOMY

**DOI:** 10.1590/0102-672020230002e1728

**Published:** 2023-05-19

**Authors:** Karina Scalabrin Longo, Thiago Bassaneze, Manuela Corrêa de Toledo Peres, Rogério Tadeu Palma, Jaques Waisberg

**Affiliations:** 1 Centro Universitário Faculdade de Medicina do ABC, Department of Gastrointestinal Surgery – Santo André (SP), Brazil.

**Keywords:** Pancreatic Fistula, Pancreaticoduodenectomy, Postoperative Complications, Prognosis, Fístula Pancreática, Pancreaticoduodenectomia, Complicações Pós-Operatórias, Prognóstico

## Abstract

**BACKGROUND:**

The high morbidity and mortality rates of pancreaticoduodenectomy are mainly associated with pancreaticojejunal anastomosis, the most fragile and susceptible to complications such as clinically relevant postoperative pancreatic fistula.

**AIMS:**

The alternative fistula risk score and the first postoperative day drain fluid amylase are predictors of the occurrence of clinically relevant postoperative pancreatic fistula. No consensus has been reached on which of the scores is a better predictor; moreover, their combined predictive power remains unclear. To the best of our knowledge, this association had not yet been studied.

**METHODS:**

This study assessed the predictive effect of alternative fistula risk score and/or drain fluid amylase on clinically relevant postoperative pancreatic fistula in a retrospective cohort of 58 patients following pancreaticoduodenectomy. The Shapiro-Wilk and Mann-Whitney tests were applied for assessing the distribution of the samples and for comparing the medians, respectively. The receiver operating characteristics curve and the confusion matrix were used to analyze the predictive models.

**RESULTS:**

The alternative fistula risk score values were not statistically different between patients in the clinically relevant postoperative pancreatic fistula and non- clinically relevant postoperative pancreatic fistula groups (Mann-Whitney U test 59.5, p=0.12). The drain fluid amylase values were statistically different between clinically relevant postoperative pancreatic fistula and non- clinically relevant postoperative pancreatic fistula groups (Mann-Whitney U test 27, p=0.004). The alternative fistula risk score and drain fluid amylase were independently less predictive for clinically relevant postoperative pancreatic fistula, compared to combined alternative fistula risk score + drain fluid amylase.

**CONCLUSION:**

The combined model involving alternative fistula risk score >20% + drain fluid amylase=5,000 U/L was the most effective predictor of clinically relevant postoperative pancreatic fistula occurrence following pancreaticoduodenectomy.

## RESUMO

**RACIONAL:**

A alta morbimortalidade da pancreaticoduodenectomia está associada, principalmente, à anastomose pancreatojejunal, a mais frágil e suscetível a complicações como a fístula pancreática pós-operatória clinicamente relevante (*clinically relevant postoperative pancreatic fistula* - CR-POPF).

**OBJETIVOS:**

O escore alternativo de risco de fístula (*alternative fistula risk score*) e os níveis de amilase do fluido de drenagem no primeiro dia pós-operatório (*first postoperative day drain fluid*) são preditores da ocorrência de fístula pancreática pós-operatória clinicamente relevante. Nenhum consenso foi alcançado sobre qual das pontuações é um melhor preditor; além disso, seu poder preditivo combinado permanece obscuro. Até onde sabemos, essa associação ainda não havia sido estudada.

**MÉTODOS:**

Este estudo avaliou o efeito preditivo do escore alternativo de risco de fístula e/ou do fluido de drenagem no primeiro dia pós-operatório em uma coorte retrospectiva de 58 pacientes após pancreaticoduodenectomia. Os testes de Shapiro-Wilk e Mann-Whitney foram aplicados para avaliar a distribuição das amostras e para comparar as medianas, respectivamente. A curva de características operacionais do receptor e a matriz de confusão foram utilizadas para analisar os modelos preditivos.

**RESULTADOS:**

Os valores do escore alternativo de risco de fístula não foram estatisticamente diferentes entre os pacientes dos grupos fístula pancreática pós-operatória clinicamente relevante e não- fístula pancreática pós-operatória clinicamente relevante (teste U de Mann-Whitney 59,5, p=0,12). Os valores de fluido de drenagem no primeiro dia pós-operatório foram estatisticamente diferentes entre os grupos fístula pancreática pós-operatória clinicamente relevante e não- fístula pancreática pós-operatória clinicamente relevante (teste U de Mann-Whitney 27, p=0,004). O escore alternativo de risco de fístula e fluido de drenagem no primeiro dia pós-operatório foram independentemente menos preditivos para fístula pancreática pós-operatória clinicamente relevante, em comparação com escore alternativo de risco de fístula + fluido de drenagem no primeiro dia pós-operatório combinados.

**CONCLUSÕES:**

O modelo combinado envolvendo escore alternativo de risco de fístula>20% + fluido de drenagem no primeiro dia pós-operatório=5.000 U/L foi o preditor mais eficaz da ocorrência de fístula pancreática pós-operatória clinicamente relevante após pancreaticoduodenectomia.

## INTRODUCTION

The high morbidity and mortality rates of pancreaticoduodenectomy, performed for the treatment of periampullary diseases, are mainly associated with pancreaticojejunal anastomosis. Of all anastomoses performed during pancreaticoduodenectomy, pancreaticojejunal anastomosis is the most fragile and susceptible to complications such as clinically relevant postoperative pancreatic fistula (CR-POPF)^7^. CR-POPF occurs in 3–45% of pancreaticoduodenectomy procedures and increases hospital stay, readmissions, and reoperations^1,2^. The occurrence of CR-POPF can also delay the start of adjuvant therapy and reduce the overall survival of patients following pancreaticoduodenectomy for malignant diseases^2,3^. Moreover, it consistently increases health care costs^2,3,4,24^. The critical factor for predicting the occurrence of CR-POPF is risk stratification by assessing patient- and procedure-related criteria and creating a fistula risk profile^4^.

The first internationally accepted definition of postoperative pancreatic fistula was established in 2005 by the International Study Group of Pancreatic Fistula^2^. It was defined as abdominal drain output of any measurable volume of fluid on and after postoperative day 3 with amylase levels in the drained fluid 3 times higher than the institutional normal serum amylase upper limit. This classification stratified patients into grade A (benign clinical course), grade B (patients at moderate risk), and grade C (critical patients requiring invasive intervention)^2^. A 2016 review of the definition of postoperative pancreatic fistula^3^ considered grades B and C as CR-POPF and renamed grade A as biochemical leak (BL), a pancreatic secretion extravasation without clinical implications. Grade B postoperative pancreatic fistula is the fistula itself and requires changes in postoperative management, including oral fasting, enteral or parenteral diet support, and antibiotic therapy in cases of infection. Moreover, it may also require an invasive intervention with percutaneous or endoscopic drainage of intra-abdominal collections. Grade C postoperative pancreatic fistula occurs when grade B postoperative pancreatic fistula is complicated with organ failure, clinical instability, need for reoperation, or death^3^. These terms are summarized in Table 1.

**Table 1:** Postoperative pancreatic fistula with its definition and grades proposed by the International Study Group of Pancreatic Surgery^3^ and the alternative fistula risk score with its parameters, calculator and risk groups^24^.

**POPF**	Abdominal drain output of any measurable volume on and after postoperative day 3 with amylase drain fluid >3 times institutional normal serum amylase upper limit
**BL**	Persistent drainage =21 days after surgery with no clinically relevant changes No therapeutic intervention required
**Grade B POPF**	Persistent drainage >21 days after surgery with clinically relevant changes Percutaneous or endoscopic drainage for abdominal collections Antibiotics for infection
**Grade C POPF**	Reoperation Organ failure Death
**CR-POPF**	POPF grade B or C
**aFRS parameters**	BMI (kg/m2) Pancreatic gland texture (soft or firm) PD size (in mm)
**aFRS calculator**	P = exp (−3.136 + 0.947 [texture] + 0.0679 [BMI] − 0.385 [PD size]) 1 + exp (−3.136 + 0.947 [texture] + 0.0679 [BMI] − 0.385 [PD size]) texture 1 = soft and 0 = firm, PD size in mm (truncated at 5)
**aFRS risk groups**	Low risk of CR-POPF (aFRS 0–5%) Intermediate risk of CR-POPF (aFRS >5–20%) High risk of CR-POPF (aFRS=20%)

POPF: postoperative pancreatic fistula; BL: biochemical leak; CR-POPF: clinically relevant postoperative pancreatic fistula; aFRS: alternative fistula risk score; BMI: body mass index; PD: pancreatic duct; P: probability; exp: exponential function.

The alternative fistula risk score (aFRS) was described in 2017^24^ and externally validated in multicenter studies^9,13,23,24,27,28^. This score considers three predictive factors of CR-POPF: pancreatic parenchyma texture, diameter of the main pancreatic duct (Wirsung's duct), and the patient's body mass index (BMI).

The aFRS classifies the risk for developing CR-POPF into three groups: low risk (aFRS 0–5%), intermediate risk (aFRS >5–20%), and high risk (aFRS >20%)^24^. These terms are summarized in Table 1. CR-POPF occurrence can also be predicted by measuring the first postoperative day drain fluid amylase (DFA)^32^.

Visual changes in the appearance of the drain fluid only become evident from postoperative day 5^22^, whereas amylase levels in the drain fluid increase already on postoperative day 1 due to its early and imperceptible extravasation through the anastomosis. Several cutoff values of DFA^1,6,8,14–17,22,25^, ranging from 90^14^ to 5,000 U/L^22^, have been described for determining the high risk of developing CR-POPF. The most cited cutoff value is 5,000 U/L^22^, thus motivating surgeons to remove the abdominal drain early when DFA values are below the aforementioned cutoff^28^.

Several studies^22,24,32^ have pointed out the importance of measuring the aFRS and DFA. With varying diagnostic values^1,6,8,14–17,22,24,25^, the determination of these scores facilitates a more individualized management, considering if the patient is at low or high risk of developing CR-POPF^4,12,18,24,30^. However, there is still no consensus on which of these scores is better and whether combining both scores would result in increased accuracy in the selection of patients with a high risk of developing CR-POPF. To the best of our knowledge, this association had not yet been studied.

## METHODS

### Study design, patients, and data collection

We carried out a retrospective cohort of 58 patients who underwent pancreaticoduodenectomy for malignant or benign periampullary disorders. All surgeries were performed in a standardized way, always by three surgeons previously designated and with consolidated experience in performing pancreaticoduodenectomy from January 2014 to December 2019 at the Department of Gastrointestinal Surgery of the ABC Medical School in São Paulo, Brazil. The study was submitted to and approved by the institution's Research Ethics Committee, on a countrywide platform (number 28434719.6.0000.0082) and was conducted in accordance with the Declaration of Helsinki. The patients’ medical records were analyzed through the institution's database, and the collected information was anonymized. A waiver for obtaining informed consent was acquired and included in the approval process by the same national platform.

The collected biodemographic data included the following information: age, gender, ethnicity, BMI, smoking history, alcoholism, and associated comorbidities (systemic arterial hypertension, diabetes mellitus, vasculopathies, and heart and lung diseases). The preoperative data also considered individual weight loss, neoadjuvant treatment, presence of obstructive jaundice, and presence of biliary prostheses.

Intraoperative events were assessed considering the surgical time, type of pancreaticojejunal anastomosis, use of transanastomotic stents, multivisceral resection, vascular reconstruction, use of abdominal drains, and transfusion of hemoconcentrated blood. The surgeon intraoperatively determined the diameter of the Wirsung's duct and the pancreatic parenchyma texture by palpation of the pancreatic gland^10^.

The postoperative progression was based on the length of hospital stay, duration of abdominal drain use, use of antibiotics and parenteral nutrition, and occurrence of pancreatic, biliary, or lymphatic fistulas. The clinical or surgical complications were assessed individually based on the need for interventions, such as percutaneous drainage of intra-abdominal collections or reoperation.

The stratification according to postoperative pancreatic fistula grades followed the International Study Group of Pancreatic Fistula guidelines^3^. The CR-POPF risk assessment used two predictive scores that had been previously described in the literature: aFRS^24^ and DFA^32^.

The aFRS was calculated using the online calculator available at www.pancreascalculator.com ^24^. The abdominal drain fluid was collected on postoperative day 1 to measure the amylase levels^32^. The original paper that describes aFRS^24^ classified patients with aFRS>20% as having a high risk for CR-POPF; we adopted this value in the present study as well. A DFA of 5,000 U/L is considered to indicate a high risk for CR-POPF^22^; we also adopted this value in the present study.

**Figure 1:**
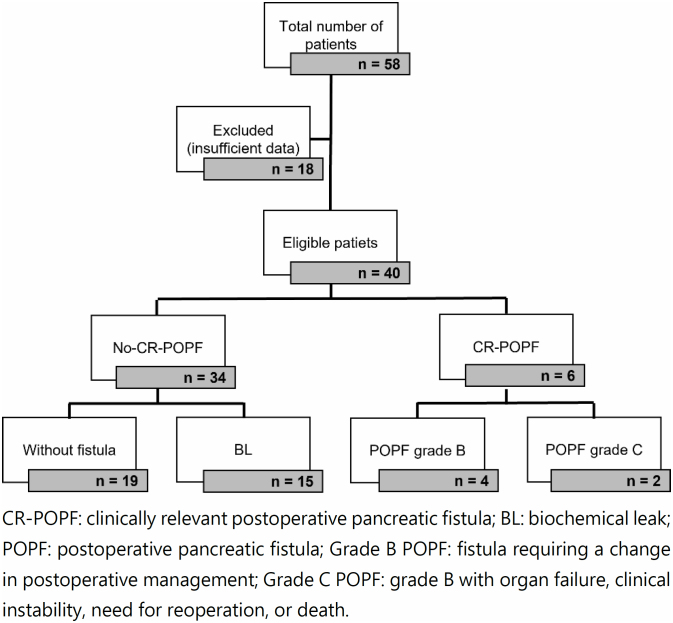
Selection of the study patients and occurrence or not of clinically relevant postoperative pancreatic fistula.

The study included adult patients of both genders who underwent elective pancreaticoduodenectomy due to malignant or benign periampullary disorders. We excluded patients with medical records having insufficient information for calculating the aFRS or without a DFA value. The medical records of 58 patients were initially reviewed, of whom 18 were excluded from the study due to insufficient information (Figure 1).

### Statistical analysis

This study assessed the correlation between aFRS and/or DFA and the occurrence of CR-POPF by performing a descriptive analysis of the data comparing the scores individually (aFRS or DFA) or combined (aFRS + DFA). The means, medians, and percentage distribution of each variable were calculated, and the corresponding boxplots were traced. The Shapiro-Wilk normality test was applied to assess the distribution of the samples and the two-tailed nonparametric Mann-Whitney test to compare the medians. The receiver operating characteristic curve and the confusion matrix were used to analyze the predictive models. Performance was determined by the metrics sensitivity, specificity, positive predictive value, negative predictive value and accuracy, and area under the curve. RStudio version 1.2.533 (RStudio, Boston, MA, USA) and Jupyterlab version 1.1.4 (Anaconda, Austin, TX, USA) were used for data analysis.

## RESULTS

### Biodemographic data

The patients were divided into two groups: those who evolved with (CR-POPF group) and without (non-CR-POPF group) CR-POPF. The biodemographic, surgical, and pathological data are summarized in Table 2.

**Table 2:** Clinical characteristics and pre-, intra-, and postoperative aspects of patients after pancreatoduodenectomy due to periampullary disorders.

Biodemographic variables	Non-CR-POPF (n=34)	CR-POPF (n=6)	Total (n=40)
Preoperative	–	–	–
Female (%)	18 (52.9)	5 (83.3)	23 (57.5)
Male (%)	16 (47.1)	1 (16.7)	17 (42.5)
Age (years)	59.4 (22–76)	58 (34–69)	59.2 (22–76)
BMI (kg/m2)	24.8 (17–35)	27.2 (21–35)	25.2 (17–35)
Ethnicity			
White (%)	22 (64.7)	4 (66.7)	26 (65)
Black (%)	12 (35.3)	2 (33.4)	14 (35)
Smoking (%)	9 (26.5)	2 (33.3)	11 (27.5)
Alcoholism (%)	4 (11.7)	0 (0)	4 (10)
Associated comorbidities (%)	19 (55.9)	4 (66.7)	23 (57.5)
Weight loss >10% (%)	18 (52.9)	2 (33.3)	20 (50)
Obstructive jaundice (%)	25 (73.5)	4 (66.7)	29 (72.5)
Intraoperative	–	–	–
Pancreatic parenchyma texture	–	–	–
Firm (%)	15 (44.1)	3 (50)	18 (45)
Soft (%)	19 (55.9)	3 (50)	22 (55)
Pancreatic duct diameter	–	–	–
=3 mm (%)	20 (58.8)	6 (100)	26 (65)
>3 mm (%)	14 (41.2)	0 (0)	14 (35)
Postoperative	–	–	–
Histopathology	–	–	–
Pancreatic head carcinoma (%)	16 (47.1)	2 (33.3)	18 (45)
Duodenal papilla carcinoma (%)	10 (29.5)	3 (50)	13 (32.5)
Chronic pseudotumoral pancreatitis (%)	3 (8.8)	0 (0)	3 (7.5)
Duodenal cancer (%)	2 (5.9)	1 (16.7)	3 (7.5)
Distal cholangiocarcinoma (%)	1 (2.9)	0 (0)	1 (2.5)
Frantz tumor (%)	1 (2.9)	0 (0)	1 (2.5)
IPMN (%)	1 (2.9)	0 (0)	1 (2.5)
Pancreatic fistula	–	–	–
CR-POPF	–	–	6 (15)
Grade B POPF (%)	0 (0)	4 (66.7)	4 (10)
Grade C POPF (%)	0 (0)	2 (33.3)	2 (5)
non-CR-POPF	–	–	34 (85)
BL (%)	15 (44.1)	0 (0)	15 (37.5)
No fistula (%)	19 (55.9)	0 (0)	19 (47.5)
Biliary fistula (%)	3 (8.8)	0 (0)	3 (7.5)
Lymphatic fistula (%)	2 (5.9)	0 (0)	2 (5)
Length of hospital stay (days)	12.9	16	13.4
Duration of drain use (days)	11.9	15.6	12.5
Percutaneous drainage (%)	0 (0)	3 (50)	3 (7.5)
Reoperation (%)	2 (5.9)	1 (16.7)	3 (7.5)
Death (%)	0 (0)	2 (33.3)	2 (5)

CR-POPF: clinically relevant postoperative pancreatic fistula; BMI: body mass index; IPMN: intraductal papillary mucinous neoplasia; BL: biochemical leak; POPF: postoperative pancreatic fistula; Grade B POPF: fistula that requires changes in postoperative management; Grade C POPF: grade B with organ failure, clinical instability, need for reoperation, or death.

All surgeries were pancreaticoduodenectomy (Whipple's procedure) and had a curative intent in cases of malignant neoplasia. Laparotomy was the access route used in all patients. The reconstruction strategy consisted of a single loop with a duct-to-mucosa pancreaticojejunal anastomosis with the placement of a transanastomotic stent. Abdominal drainage was performed with a silicone tubulolaminar drain placed near the pancreaticojejunal anastomosis. Multivisceral and vascular resection was performed in 1 (2.5%) case. The mean surgical time was 338.5±123.42 min (150–630 min). The mean estimated intraoperative blood loss was 570±427.71 mL (50–1500 mL). The Wirsung's duct diameters were 3 mm in 26 (65%) patients and >3 mm in 14 (35%). The pancreatic parenchyma texture was considered firm in 18 (45%) patients and softened in 22 (55%).

Postoperatively, biliary fistula was observed in 3 (7.5%) cases, of which 2 were surgically treated and 1 was conservatively treated, and fistulas were resolved in all cases. Lymphatic fistula was observed in 2 (5%) cases, both of which progressed favorably after clinical treatment. No drugs were used to prevent the appearance of a pancreatic fistula or decrease pancreatic secretion volume.

There were 6 (15%) cases of pancreatic fistula, of which 4 (10%) were with grade B and 2 (5%) with grade C. CR-POPF was not observed in 34 (85%) cases, of which 15 (37.5%) had BL and 19 (47.5%) did not have any type of fistula. The 2 patients with grade C died from multiple organ failure, 1 on postoperative day 6 due to acute renal failure and systemic inflammatory response syndrome (SIRS) and the other on postoperative day 25 after reoperation. Of the 4 patients with grade B postoperative pancreatic fistula, 3 underwent percutaneous drainage of abdominal collections and 1 followed conservative clinical treatment, all of them with good progression.

The mean length of hospital stay was 16 and 12.9 days in the CR-POPF and non-CR-POPF groups, respectively (Mann-Whitney U test; p>0.05). The mean duration of abdominal drain use was 15.6 and 11.9 days in the CR-POPF and non-CR-POPF groups, respectively (Mann-Whitney U test; p>0.05).

### Correlation between alternative fistula risk score and clinically relevant postoperative pancreatic fistula

**Figure 2:**
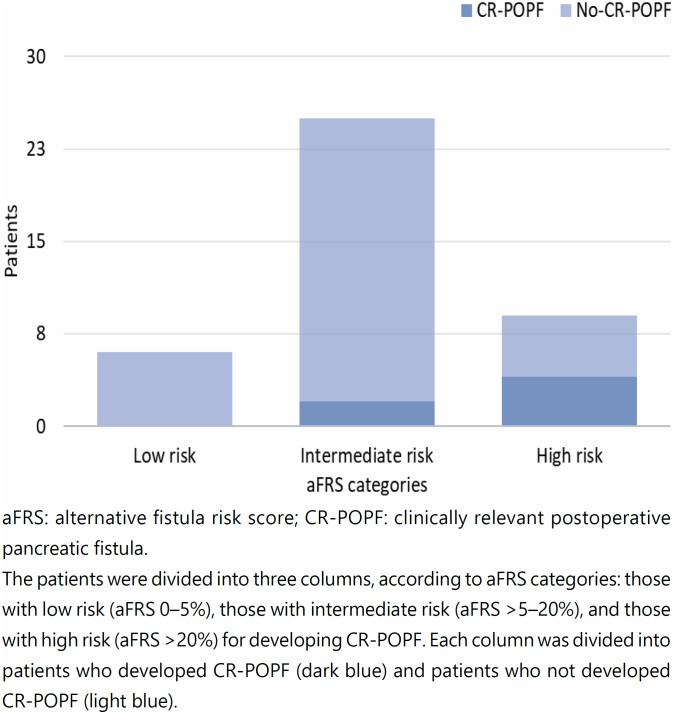
Analysis of the distribution of aFRS categories in relation to the occurrence of CR-POPF.

The mean aFRS was 22.38 and 12.23% in the CR-POPF and non-CR-POPF groups, respectively. We classified 6 (15%) patients as low (aFRS 0–5%), 25 (62.5%) as intermediate (aFRS >5–20%), and 9 (22.5%) as high risk (aFRS >20%) for CR-POPF. Considering aFRS for risk stratification of the sample population, none of the patients at low, 2 (8%) of the intermediate, and 4 (44.4%) of the high risk developed CR-POPF (Figure 2).

**Figure 3:**
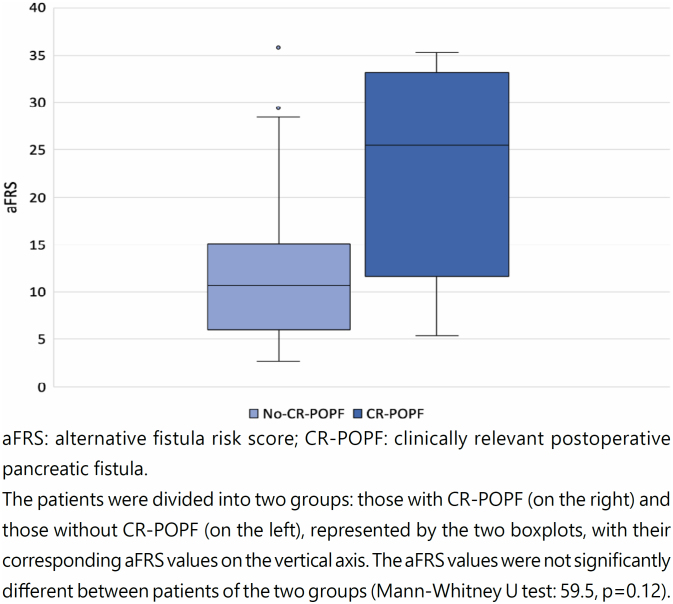
Analysis of the distribution of aFRS values in relation to the occurrence of CR-POPF.

The analysis of the variability of aFRS in relation to the presence of CR-POPF demonstrated that the median aFRS was higher in the CR-POPF group compared to that in the non-CR-POPF group (Figure 3).

The distribution of the aFRS values was nonparametric (p<0.05; Shapiro-Wilk normality test). There were no significant differences between the CR-POPF and non-CR-POPF groups (Mann-Whitney U test: 59.5, p=0.12).

### Correlation between day drain fluid and clinically relevant postoperative pancreatic fistula

The mean DFA was 19,082 and 2,388 U/L in the CR-POPF and non-CR-POPF groups, respectively. Considering DFA for risk stratification of the sample, 7 (17.5%) patients presented values 5,000 U/L, 5 (71.4%) of whom evolved with CR-POPF. Contrarily, 33 (82.5%) patients presented values <5,000 U/L, 1 (3%) of whom evolved with CR-POPF. It was observed that the mean DFA of the 2 groups had very different magnitudes, indicating a higher discriminatory power of this variable.

**Figure 4:**
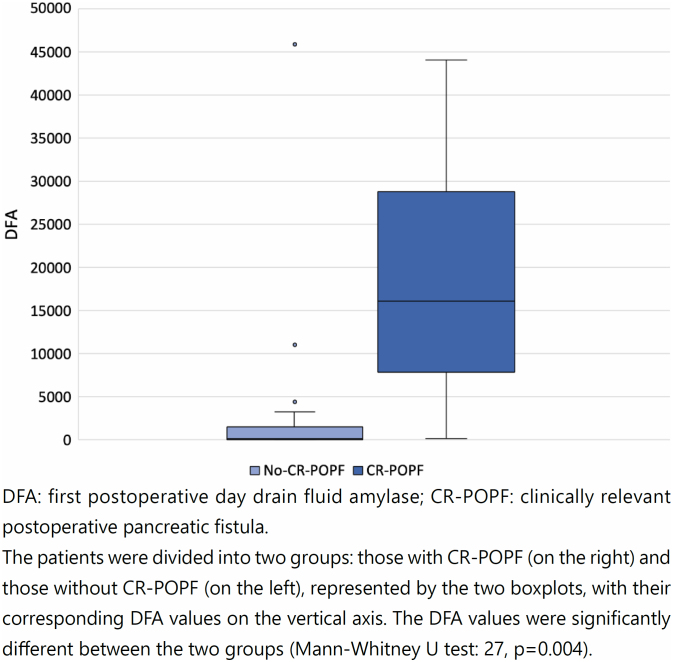
Analysis of the distribution of DFA values in relation to the occurrence of CR-POPF.

The analysis of the variability of DFA in relation to the presence of CR-POPF demonstrated that the DFA medians differed significantly based on the presence or absence of CR-POPF (Figure 4).

Both aFRS and DFA had higher median values in the CR-POPF compared to the non-CR-POPF group. However, unlike the previous analysis (aFRS and CR-POPF; Fig. 3), it was possible to completely separate the data in the boxplots in this analysis (DFA and CR-POPF; Figure 4), which corroborates the strong discriminatory power of DFA.

The distribution of DFA values was nonparametric (p<0.05; Shapiro-Wilk normality test). There were differences between the CR-POPF and non-CR-POPF groups (Mann-Whitney U test: 27, p=0.004). This indicates that DFA has a greater discriminatory power for the occurrence of CR-POPF compared to aFRS.

### Comparing the predictive models

The maximum accuracy of aFRS alone was 0.85, with a value of 29.4%. A sensitivity of 0.33 and a specificity of 0.94 (positive predictive value=0.50, negative predictive value=0.88) were observed for that cutoff value. Using the cutoffs provided in the literature, for aFRS=5% and aFRS=20%, sensitivities were 1.00 and 0.66, specificities were 0.17 and 0.85, positive predictive values were 0.17 and 0.44, negative predictive values were 1.00 and 0.93, and accuracies were 0.30 and 0.82, respectively.

The maximum accuracy of the DFA alone was 0.92, with a value of 4,377 U/L. A sensitivity of 0.83 and a specificity of 0.94 (positive predictive value=0.71, negative predictive value=0.96) were observed for that cutoff value. The metrics were similar for DFA=5,000 U/L. An “accuracy freeze” occurred for DFA values above 4,377 U/L, in which the values of the confusion matrix coincided with the cutoff values obtained through the accuracy analysis and the cutoff above this value did not allow for the definition of new cutoff values.

For the combined model of aFRS >20% + DFA=5,000 U/L, the sensitivity, specificity, positive predictive value, negative predictive value, and accuracy were 0.66, 1.00, 1.00, 0.94, and 0.95, respectively.

The values of the metrics of the predictive models are summarized in Table 3.

**Table 3:** Metrics calculated for each predictive model, with the variables singly or combined: aFRS, DFA, and aFRS + DFA.

Metrics	aFRS =5%	aFRS >20%	DFA =5,000 U/L	aFRS >20% + DFA =5,000 U/L
Se	1.00	0.66	0.83	0.66
Sp	0.17	0.85	0.94	1.00
PPV	0.17	0.44	0.71	1.00
NPV	1.00	0.93	0.96	0.94
Accuracy	0.30	0.82	0.92	0.95

Se: sensitivity; Sp: specificity; PPV: positive predictive value; NPV: negative predictive value.

**Figure 5:**
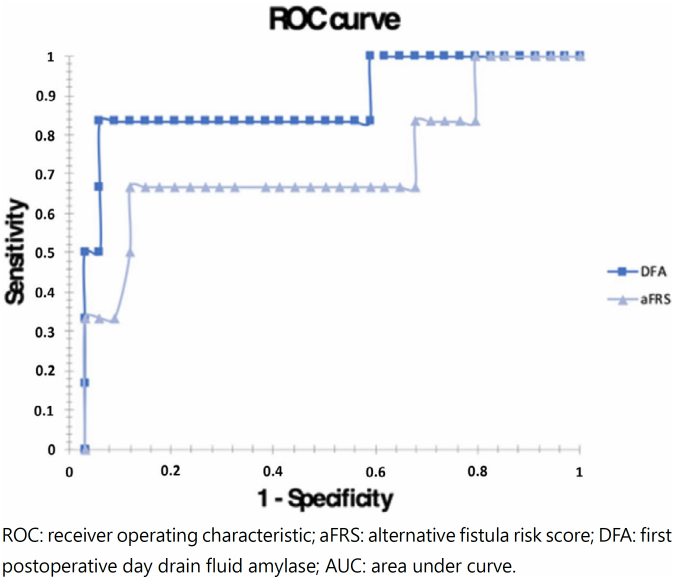
ROC curve comparing the different cutoff values for aFRS and DFA and the corresponding AUC. AUC (aFRS) 0.71; AUC (DFA) 0.86.

The receiver operating characteristic (ROC) curve was plotted for aFRS and DFA to analyze the different cutoff values and find the points of the greatest specificity and sensitivity to differentiate the CR-POPF from the non-CR-POPF group (Figure 5). The area under the curve (AUC) was obtained from the ROC curve for each predictive model: area under the curve for aFRS=0.71 and DFA=0.86.

## DISCUSSION

CR-POPF is one of the most serious complications and is responsible for the high morbidity and mortality rates of pancreaticoduodenectomy^26^. The morbidity rates range from 30 to 50% in large reference centers; however, the mortality rates have been reduced to less than 5% due to recent advances in perioperative management^2,3,22^. The clinical impact of CR-POPF leads to significantly higher health costs by increasing hospital stay and the need for invasive interventions^4,22,24^.

This study assessed the association between aFRS and DFA and the occurrence of CR-POPF and compared the models for predicting the occurrence of CR-POPF.

The incidence of CR-POPF in the present study was 15%, which is in agreement with that reported in the literature^2,3,4,24^. The CR-POPF group showed a trend toward smaller Wirsung's duct diameters and greater BMIs, which are parameters that are used for calculating aFRS^24^.

The Wirsung's duct diameter and the pancreatic parenchyma texture are criteria considered subjective since these measurements are intraoperatively determined by the surgeon's autonomy. However, studies^10^ suggest that the determination of these criteria by experienced surgeons is accurate and intraoperative measurements can correlate with corresponding findings on preoperative images (three-phase computed tomography or nuclear magnetic resonance)^11^.

Mungroop et al.^24^ have demonstrated that sensitivity and specificity are improved by using the aFRS for predicting the occurrence of CR-POPF compared to the original fistula risk score (FRS). Moreover, the aFRS has become more feasible in clinical practice as it does not use the histopathological examination report and the measurement of intraoperative bleeding, information that is required for calculating the original FRS^4^. Furthermore, it is difficult for surgeons to calculate the original FRS since the measurement of intraoperative bleeding may not be accurate and the histopathological report is often only available in the late postoperative period^24^.

The calculation of the aFRS is inherent to the patient and its use in the literature has shown to be of great value for screening patients, especially those at low risk of CR-POPF occurrence^9,13,24,27,28^. The lower values (aFRS=5%) in the present study, although not very accurate, have a high sensitivity and negative predictive value. As predicted by the low-risk score (aFRS=5%), none of the patients in the present study evolved with CR-POPF. Lao et al.^13^ retrospectively evaluated the external validation of aFRS in 370 patients who underwent pancreaticoduodenectomy and found a low accuracy (0.46) and high sensitivity (0.92) and negative predictive value (0.94). The present study corroborated these results and thus suggests the use of this indicator for selecting patients at low risk for CR-POPF.

The correlation of the aFRS alone with the CR-POPF showed a trend toward fistula occurrence since the aFRS value increased, which agrees with results found in the literature^9,13,24,27,28,29^.

DFA measurement is a simple procedure; however, it requires the presence of the abdominal drain. Molinari et al.^22^ correlated the DFA with the occurrence of CR-POPF and encouraged the strategy of early removal of the abdominal drain depending on DFA values. The cutoff value provided in the literature^1,6,8,14–17,22,25^ ranged from 90^14^ to 5,000 U/L^22^. Giglio et al.^8^ performed a meta-analysis with 13 studies and 4,416 patients to assess the accuracy of 11 different DFA cutoff values as predictors of CR-POPF and found the highest specificity for the value of 5,000 U/L.

The correlation between DFA and CR-POPF showed a trend toward fistula occurrence as DFA values increased, which is in agreement with findings from other studies^1,6,8,14–17,22,25^. In the present case series, the DFA showed a high discriminatory power, since the means of the two groups had very different magnitudes. The DFA boxplot visually showed its strong predictive power by a significant independent separation of data. Moreover, the accuracy of the DFA for predicting the occurrence of CR-POPF was higher than that of the aFRS when the scores were analyzed individually. For these reasons, the DFA alone proved to be a strong predictive parameter for CR-POPF occurrence, and with a greater discriminatory power compared to the aFRS.

The most accurate model in the present study for predicting CR-POPF occurrence was aFRS >20% + DFA=5,000 U/L. There was an increase in accuracy when the scores were combined. Notably, this combination used the two values of aFRS^24^ and DFA^22^ obtained in the literature for determining the high risk for CR-POPF. The combination of these two scores is of great interest since their methods and advantages for predicting CR-POPF seem broad and overlapping. To the best of our knowledge, this association between aFRS and DFA had not yet been studied.

Therefore, using the aFRS and DFA, it was possible to predict CR-POPF occurrence intraoperatively and on postoperative day 1, respectively, with greater accuracy for the combined aFRS + DFA. Although our sample is small, this information may help in further studies to identify patients at high risk for CR-POPF (aFRS >20% and DFA=5,000 U/L) who may be candidates for more rigorous postoperative monitoring, and patients at low risk for CR-POPF (aFRS=20% and DFA <5,000 U/L) who may be the candidates for fast-track protocols such as early return to normal diet, removal of the abdominal drain, and discharge. Nevertheless, our results need to be confirmed with prospective clinical studies^5,12,19–21,25,28,30,31^.

Kawai et al.^12^ argued that early discharge (around postoperative day 5) can be safe and economically beneficial for patients with good progression following pancreaticoduodenectomy, while others encourage the omission of the prophylactic drain in patients with low risk of CR-POPF, as well as its early removal when the DFA is <5,000 U/L^5,12,18–21,25,28,30,31^. However, most institutions still use the abdominal drain routinely and remove it on or after postoperative day 7^12^.

Postoperative management is planned based on existing complications, which affect the length of hospital stay and the patient's morbidity and mortality^22^. Regarding the length of hospital stay and duration of abdominal drain use, the present case series showed a trend for the CR-POPF group to stay longer in the hospital compared to the non-CR-POPF group, and an association between the decision to remove the drain and the decision to discharge.

This study was limited by the small sample size. Moreover, it was a retrospective and observational cohort study. The association between aFRS and DFA may help differ the patients with low risk from those with high risk of developing CR-POPF, but further studies with a larger number of patients are required to confirm the possible prediction of CR-POPF occurrence with the combined use of the scores.

## CONCLUSION

The data obtained in this study demonstrated that the combined use of the aFRS and first postoperative day DFA increased the accuracy for predicting CR-POPF in patients who underwent pancreaticoduodenectomy. Prospective studies with a larger number of cases are required to confirm these results.
